# Walking balance is mediated by muscle strength and bone mineral density in postmenopausal women: an observational study

**DOI:** 10.1186/s12891-018-2000-3

**Published:** 2018-03-15

**Authors:** S. C. Ibeneme, C. Ekanem, A. Ezuma, N. Iloanusi, N. N. Lasebikan, O. A. Lasebikan, O. E. Oboh

**Affiliations:** 1 Department of Medical Rehabilitation, Faculty of Health Sciences, University of Nigeria, Enugu Campus, Enugu, Nigeria; 2 Exercise Immunology/Palliative Care Unit, Department of Physiotherapy, University of Nigeria Teaching Hospital, Ituku Ozalla, Enugu, 400001 Nigeria; 3 Department of Radiation Medicine, University of Nigeria Teaching Hospital , Enugu, 4000001 Nigeria; 4 Department of Orthgopaedic Surgery, National Orthopaedic Hospital, Enugu, 4000001 Nigeria; 5 Department of Radiotherapy, University of Benin Teaching Hospital, Benin, Edo Nigeria; 6 Clinical Trial Consortium, University of Nigeria, Nsukka Enugu, Nigeria; 7 UNIRED Research Group, Hochschule Hannover - University of Applied Sciences and Arts, Hannover, Germany

**Keywords:** Muscle strength, Body composition, Bone mineral density, Walking balance, Postmenopausal women

## Abstract

**Background:**

Depletion of ovarian hormone in postmenopausal women has been associated with changes in the locomotor apparatus that may compromise walking function including muscle atrophy/weakness, weight gain, and bone demineralization. Therefore, handgrip strength (HGS), bone mineral density (BMD) and body composition [percentage body fat mass (%BFM), fat mass (FM), Fat-free mass (FFM) and body mass index (BMI)], may significantly vary and predict WB in postmenopausal women. Consequently, the study sought to 1. Explore body composition, BMD and muscle strength differences between premenopausal and postmenopausal women and 2. Explore how these variables [I.e., body composition, BMD and muscle strength] relate to WB in postmenopausal women.

**Method:**

Fifty-one pre-menopausal (35.74 + 1.52) and 50 postmenopausal (53.32 + 2.28) women were selected by convenience sampling and studied. Six explanatory variables (HGS, BMD, %BFM, FFM, BMI and FM) were explored to predict WB in postmenopausal women: Data collected were analyzed using multiple linear regression, ANCOVA, independent t-test and Pearson correlation coefficient at *p* < 0.05.

**Result:**

Postmenopausal women had higher BMI(t = + 1.72; *p* = 0.04), %BFM(t = + 2.77; *p* = .003), FM(t = + 1.77; *p* = 0.04) and lower HGS(*t* = − 3.05; *p* = 0.001),compared to the premenopausal women. The predicted main effect of age on HGS was not significant, F(1, 197) = 0.03, *p* = 0.06, likewise the interaction between age and %BFM, F(1, 197) = 0.02, *p* = 0.89; unlike the predicted main effect of %BFM, F(1, 197) = 10.34, *p* = .002, on HGS. HGS was the highest predictor of WB (*t* = 2.203; β=0.3046) in postmenopausal women and combined with T-score right big toe (Tscorert) to produce R^2^ = 0.11;F (2, 47)=4.11;*p* = 0.02 as the best fit for the predictive model. The variance (R^2^) change was significant from HGS model (R^2^ = 0.09;*p* = 0.03) to HGS + Tscorert model (R^2^ = 0.11;p = 0.02). The regression model equation was therefore given as: WB =5.4805 + 0.1578(HGS) + (− 1.3532) Tscorert.

**Conclusion:**

There are differences in body composition suggesting re-compartmentalization of the body, which may adversely impact the (HGS) muscle strength in postmenopausal women. Muscle strength and BMD are associated with WB, although, only contribute to a marginal amount of the variance for WB. Therefore, other factors in addition to musculoskeletal health are necessary to mitigate fall risk in postmenopausal women.

## Background

Walking balance (WB) was operationalized as walking in an upright position without the elicitation of equilibrium reactions/wavering [1]. This mechanism which is operationally deployed to stabilize the body can be compromised from the consequent effects of aging such as weakness in bone, changes of body composition [increased percentage body fat mass (%BFM), increased fat mass(FM), decreased Fat-free mass(FFM) and increased body mass index (BMI)] and muscle status in postmenopausal women [2–4]. The handgrip strength (HGS) provides a measure of the changes in muscle status, general health status and serves as a predictor of other health conditions likely to impact WB, although it is not the causative factor [5, 6]. Importantly, HGS has a positive relationship with bone mineral density (BMD) in postmenopausal women [7]. Invariably, there could be a link between body composition, HGS, BMD and WB, which needs to be considered when making clinical decisions about menopause. Therefore, the objectives of the study are to 1. Explore body composition, BMD and muscle strength differences between premenopausal and postmenopausal women and 2. Explore how these variables [I.e., body composition, BMD and muscle strength] relate to WB in postmenopausal women.

Menopause is a transition period, which is a function of aging and usually occurs in the age range of 42–58 years [8, 9]. It is characterized by psychosocial reorientation(mood disorders - anxiety and depression related to body image and self-esteem), sociological changes (due to loss of reproductive function, diminished sexual function and perception of self-worth/relevance in relationships) and anxiety-related physiological variations (symptoms – such as sweating, and hot flushes resulting in increased muscle fatigue and feelings of lethargy) [9, 10] that accompany the depletion of the ovarian hormones [11]. Such symptoms as depression and fatigue may negatively impact the physical activity level of postmenopausal women, especially walking function. In addition, postmenopausal estrogen decrease has been linked with several physiologic changes in the body composition including increase in fat mass, increased visceral adiposity and BMI [12] Overall, there is a decrease in lean body mass or FFM [13], BMD and HGS [14, 15]. Meanwhile, an increase in the BMI or body mass (BM) at the same or constant height (ht), implies a relative increase in body weight (load) or BM since BMI = BM/ ht^2^; BM = FM + FFM [16]. A concomitant decrease in FFM and HGS (muscle strength) in postmenopausal women not only suggests a recompartmentalisation/change in body composition, but also a mismatch between body weight (load) and muscle strength available to influence it, which is a common feature of fallers [17, 18]. Invariably, with increasing weight gain, an individual with weakened or fatigued muscles, which is common in postmenopausal women [9–11, 14, 15], will likely be progressively unstable and prone to fall in upright stance [17, 18]. Therefore, a match between body weight and available muscle strength may be a critical factor for postural stability, which may have implications for WB in postmenopausal women. This may help to explain why body weight is a predictor of postural stability [19]. In this context, postural stability was operationalized as an individual’s ability to maintain a state of equilibrium without swaying, staggering or reeling and thus minimize the tendency to fall. Invariably, factors that impact postural stability such as body weight, and muscle strength, should have a predictive effect on WB. It was, therefore, predicted that body composition, HGS, and BMD might explain variance in WB among postmenopausal women.

## Method

Five hundred and sixty-four consenting female staff of the University of Nigeria Teaching Hospital (UNTH), Ituku/Ozalla, Enugu, who were identified from the hospital telephone directory were targeted in a cross-sectional observational study. The UNTH Enugu was selected because many studies have been done on menopause in this hospital community, [2] and therefore allows for contextual interpretations of the findings relative to previous studies. Fisher’s equation was used to determine the sample size for the study with reference to the estimated national menopause prevalence of 3.6% giving a sample size of 58 participants. This is reasonable, since 0–3 patients per month seen by gynecologists across Nigeria, presented with symptoms of menopause [20], and 1% prevalence rate of premature menopause have been previously reported [21].

**Fig. 1:**
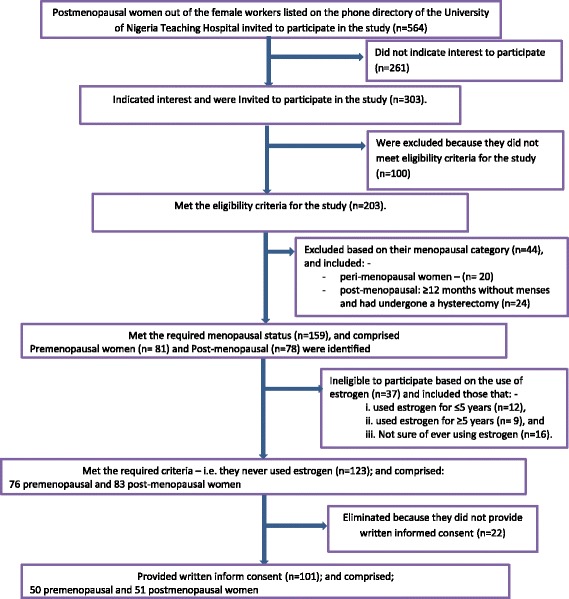
Design and flow of participants through the study

The 564 female workers, on the hospital’s phone directory, were contacted via email that explained the purpose of the study while soliciting for their participation (Fig. 1). A follow-up contact phone calls were made by 4 research assistants using the hospital’s phone directory and only 303 female workers with right-handed/limb dominance, indicated willingness to participate in the study and were enlisted. The participants that met the eligibility criteria, for the study, were subjected to various physical assessments likewise their walking balance. Five inclusion criteria were applied as follows: - At least 1-year regular menstrual cycle, prior to the study, for women at pre-menopause, Non-menstruation for at least one year; for in postmenopausal women, 3) No history of other diseases of metabolic, neurologic or orthopedic nature, 4) Age not < 25 but not > 64 years; and 5) Only right-handed (i.e. left cerebral dominance) individuals were recruited, This is important, because handedness may influence HGS and subsequent skeletal loading/traction on the bone during physical activity may influence the BMD [22]. Moreover, since right-hand dominance was found to be at 92.6% in boys and at 91.9% in girls [23], it will be easier to recruit equivalent groups of pre-and postmenopausal women. Therefore, lack of consideration for handedness may be a reasonable threat to internal validity of the study and may not provide a reference or basis that may be required when comparing the findings of the study with similar works in the literature.

Exclusion criteria: -

1. Individuals on bisphosphonates, steroids or other drugs which could influence bonemetabolism/mineral density [24], were not eligible, and
2. Individuals with comorbid conditions that may affect bone health (e.g. CHF, renal failure and cancer) [25–27] were not eligible.

This population was further categorized based on their menopausal status, using the information they provided in a self-reported proforma. According to Ibeneme et al. [2], menopausal statuses was classified into 4: 1) pre-menopausal: regular menstrual cycles; 2) peri-menopausal: ≥3 months without menses, large changes in cycle length and hot flashes; or 3) post-menopausal: ≥12 months without menses and had undergone a hysterectomy. or 4) post-menopausal: ≥12 months without menses and had not undergone a hysterectomy. Based on this classification, 81 premenopausal women and 78 postmenopausal women, who have not undergone hysterectomy, were identified and required to further indicate whether they have used estrogen. From the data they provided, they were subsequently categorized into 4 groups: i. Never used estrogen, ii. Used estrogen for ≤5 years, iii. Used estrogen for ≥5 years and iv. Not sure of ever using estrogen. Only those who never used estrogen were drawn into the sample comprising 76 premenopausal women and 83 postmenopausal women. Of this number, only those that provided signed written informed consent were selected for the study, and comprised 50 premenopausal and 51 postmenopausal women, respectively.

The study process comprised three stages: obtaining informed consent, physical assessment and measurement of walking balance. The test instruments were: -

i. Fat Loss Monitor with Scale (OMRON® HBF-400): - The equipment measures Weight: 0.0 lb. to 330.0 lbs. in 0.2 lb. increments; %BFM: 5.0 to 60.0% in 0.1% increments; and BMI: 7.0 to 90.0 in 0.1 increment. DEXA (Dual Energy X-Ray Absorptiometry) has been the established method for accurate evaluation of body composition. OMRON has used research information from several hundred people from 10 to 80 years of age using the DEXA method to develop the formula by which the Fat Loss MONITOR with Scale works. The body fat mass and body fat percentage are calculated by a formula that includes five factors: electrical resistance, height, weight, age and gender.
ii. Hand Dynamometer (Lafayette, model 78,010, USA). This equipment features a dual- scale readout that displays isometric grip force from 0 to 200 pounds (0–100 kg). The HGS was read off to the nearest 1.0 kg.
iii. Stadiometer: - The height was read off and recorded to the nearest 1.0 cm at the vertex of the head with the participants standing barefoot.
iv. Measuring tape (butterfly brand, made in China): A 15 m long measuring tape was used to mark out the required 3-m distance on the ground.
v. Stop clock (Heurer brand, made in Germany). The stop clock was used to measure the walk time required to cover the 3-m distance and was read off to the nearest second.
vi. Weighing scale (Hana bathroom scale, made in China) – This was used to obtain the weight of the subjects. The body weight was read off to the nearest 1.0 kg.

### Data collection

The 4 research assistants were trained on specific assessment procedure to ensure consistency in measurement and minimize inter-observer errors. Thus, one assistant was trained specifically for any of the four assessment tasks, namely i. Measurement of the BMD, ii. HGS, iii. Body composition [%BFM, FFM, BMI and FM], height, and, iv. WB. A pilot study was subsequently conducted at the University of Nigeria, Enugu Campus to ensure that the research assistants were acquainted with the test instruments and relevant operational procedures prior to the study.

Procedures for the study were explained to the participants and only those that gave their written informed consent were involved in the study. Participants’ anthropometric data were measured, including weight and height. The data generated were imputed into the bio-impedance electronic Fat Loss Monitor with Scale (OMRON® HBF-400) for each individual. They were requested to stand barefoot on the equipment with their feet, clean and dry. Participants were asked to position their feet on the electrodes and make sure each of the heels is positioned on a heel electrode, with weight evenly distributed on the measurement platform. They were asked to remain still and not move until the measurement is complete and readings of their body compositions were displayed on the LCD screen. Thereafter, Hume’s formula [(0.29569*W) + (0.41813 * HT) – 43.2933. Where W = weight, HT = height] [28, 29] was used to calculate the lean muscle mass or FFM.

The HGS was subsequently measured using a dynamometer as prescribed by American Society for Surgery of the Hand and the American Society of Hand Therapists, which is described in detail elsewhere [30]. The grip strength was determined with the subjects in a sitting position, and with the shoulder adducted and neutrally rotated, while the elbow was flexed to 90 degrees, and the forearm and wrist held in a neutral position. The two arms (immovable and moveable) were adjusted to zero from the onset. The subjects were then asked to grip the equipment and sustain it for 1 min. The moveable arm was displaced according to the strength of the grip and came to rest at the point of maximum grip strength. The difference between the position of the immovable arm and the position of the moveable was determined as the value of the hand grip strength. Three trials were carried out for right dominant hand and the mean was determined and recorded.

Subsequently, the participants’ WB was assessed using the ‘Timed Up & Go Test’ [31]. Each participant was asked to sit in a chair and from which they were required to rise with the command - “ready, set, go.” Then, the participants were required to walk forward over a 3-m (10 ft) distance and back to the chair. The Stop clock was stopped and the time was recorded on completion of the task. The BMD of the right and left Hallux (i.e. big toe), in right-handed individuals, was measured using the Xrite 331C densitometer. To Null the instrument, the following steps apply 1. Remove film from reading area.2. Lower the reading arm. Press the “NULL” button and hold while pressing the “MEASURE” button. 3. Hold both buttons down until the reading on the display has stabilized. Thereafter, the absolute density was measured as follows: 1. Null the instrument as previously described., 2. Center the film area in question directly over the aperture under the reading arm.3. Lower the reading arm. Press the “MEASURE” button and hold for a few seconds until the lamp goes out. 4. Remove pressure on the MEASURE button so that the reading arm rises. The density measured will be displayed until the button is pushed again. To compare the density readings, the following steps apply 1. Place reference film over the aperture. Null the instrument as previously described. 2. Place the film to be compared over the aperture and measure the density. This measurement is the difference between the reference film density (2.99dens) and the compared film density. A minus (−) display indicates the compared film is a lower density. Normal: Bone density is within 1 SD (+ 1 or − 1) of the young adult mean. Low bone mass (osteopenia): Bone density is 1 to2.5 SDs below the young adult mean (− 1 to − 2.5 SD). Osteoporosis: Bone density is 2.5 SDs or more below the young adult mean (less than − 2.5 SD) [32].

### Data/statistical analysis

Multiple linear regression analysis was conducted to assess the effect of the six explanatory variables (HGS,, %BFM, FFM, BMI, FM and T-score of the right big toe(T-scorert) on WB, in postmenopausal women, after adjustment for age [33], smoking status [34], level of physical activity [35], alcohol intake, [36, 37] vitamin K intake, [38] comorbid conditions (e.g. chronic heart failure, renal failure and cancer) [25–27] and drug interactions (e.g. amiodarone, statins, non-steroidal anti-inflammatory drugs, antiplatelet agents) [39, 40]. Three explanatory models were proposed to explain the variance in WB among postmenopausal women, namely, i. HGS model, ii. HGS + %BFM model and iii. HGS + %BFM + T-scorert model. Based on an ordinary least square (OLS) regression analysis of these models, nonsignificant interactions and predictors were removed. The explanatory variables in a multiple linear regression equations should be independent of one another [41]. If two or more explanatory variables are correlated, that is, if their regression lines are parallel or “collinear,” then they are not independent. Collinear variables add much the same information to the model, so only one is needed. The variable with the strongest relationship with the response variable (WB) should be considered for inclusion in the final model. Analysis of covariance (ANCOVA) was used to predict the main effects of age on HGS and its interaction effects with %BFM. Faculty Vassar computational website software was used for the data analysis. Alpha was set at *p* < 0.05.

## Results

The design and flow of participants through the study is shown in Fig. 1. The results of this study showed that (Table 1) the postmenopausal women were significantly (*p* < 0.05) older, shorter in height and with greater BMI than the premenopausal women. In contrast, there was no significant (*p* > 0.05) difference in body weight of the postmenopausal women compared to the premenopausal women. Analysis of the body composition (Table 2) showed that the postmenopausal women had significantly (*p* < 0.05) higher %BFM and FM compared to the premenopausal women. However, there was no significant difference (*p* < 0.05) in the FFM between both groups. Furthermore, the premenopausal women had significantly (*p* < 0.05) higher HGS than the postmenopausal women. The predicted main effect of age on HGS was not significant, F(1, 197) = 0.03, *p* = 0.06, unlike the predicted main effect of %BFM, F(1, 197) = 10.34, *p* = .002. The interaction between age and %BFM were also not significant, F(1, 197) = 0.02, *p* = 0.89. Test for homogeneity of regressions was not significant, F(3, 197) = 0.76, *p* = 0.52. Overall, there was no significant (*p* > 0.05) group difference in WB when postmenopausal women were compared to the premenopausal women (Table 3). Similarly, T-score (Table 4) for the right and left toe did not differ significantly (*p* > 0.05) in the postmenopausal women compared to premenopausal women.

**Table 1:** Anthropometric Characteristics of premenopausal (*N* = 51) and Postmenopausal (*N* = 50) women

Menopausal status	Age		Height		Weight		Body mass index	
	X ± SD Range		X ± SD Range		X ± SD Range		X ± SD Range	
Pre-menopause	35.74 ± 1.52	27–49	1.63 ± 2.33	1.47–1.8	73.09 ± 4.52	50.6–100.9	27.47 ± 1.62	18.6–42.1
Post-menopause	53.32 ± 2.28	42–71	1.60 ± 2.05	1.35–1.76	75.01 ± 4.74	47.3–129	29.49 ± 1.72	18.8–40.1
Mean difference	17.57 ± 2.70		−3.45 ± 3.08		1.92 ± 6.49		2.02 ± 2.34	
t-value	+ 12.93		−2.23		+ 0.59		+ 1.72	
df	99		99		99		99	
p-value one tailed	< 0.0001***		0.01*		0.28		0.04*	

*Indicates significance at *p* < 0.05; ***indicates significance at *p* < 0.0001

**Table 2:** Variations in body composition and handgrip strength in premenopausal (*N* = 51) and postmenopausal (*N* = 50) women

Menopausal status	%Body fat	Fat mass	Fat-free mass	Handgrip strength
Pre-menopause	22.41 ± 2.16	17.32 ± 2.49	45.92 ± 1.86	35.10 ± 3.80
Post-menopause	26.22 ± 6.05	20.42 ± 2.51	46.55 ± 2.17	27.57 ± 3.18
Mean difference	3.82 ± 3.89	3.10 ± 3.50	−0.62 ± 4	−7.52 ± 4.91
t-value	+ 2.77	+ 1.77	−0.44	−3.05
df	99	99	99	99
p-value one tailed	0.0033**	0.04*	0.3304	0.0015**

*Indicates significance at *p* < 0.05; **indicates significance at *p* < 0.001

**Table 3:** Variations in Static balance and walking balance in in premenopausal (*N* = 51) and Postmenopausal women (*N* = 50)

Menopausal status	Static balance	Walking balance
Pre-menopause	3.30 ± 0.52	9.76 ± 0.47
Post-menopause	4.65 ± 1.33	10.88 ± 5.80
Mean difference	1.35 ± 1.40	1.12 ± 4.43
t-value	+ 1.92	+ 1.32
df	99	99
p-value one tailed	0.03*	0.09

*Indicates significance at *p* < 0.05

**Table 4:** Variations in bone mineral density and Tscore in premenopausal (*N* = 51) and Postmenopausal (*N* = 50) women

Menopausal status	Right Toe		Left Toe	
	BMD	T Score	BMD	T Score
Pre-menopause	2.38 ± 0.22	−0.59 ± 0.22	2.20 ± 0.19	−0.61 ± 0.19
Post-menopause	2.19 ± 0.29	−0.78 ± 0.29	2.39 ± 0.18	−0.76 ± 0.19
Mean difference	−0.19 ± 0.36	−0.18 ± 0.36	− 0.19 ± 0.26	−0.15 ± 0.26
t-value	−1.04	− 1.00	− 1.49	− 1.16
df	99	99	99	
p-value one tailed	0.15	.16	0.07	0.12

BMD = bone mineral density; Tscore = Standardized bone mineral density score relative to sex and age

Basic descriptive statistics and regression coefficients are shown in Tables 5, 6 and 7. For body composition, multicollinearity of the explanatory variables was evident since the FFM, FM, BMI, and %BFM were correlated. Given that the %BFM is the measure of body composition with the strongest correlation (*r* = 0.061) with WB, FFM (*r* = 0.02), FM (*r* = 0.055) and BMI (*r* = 0.03) were removed from the model. Thus, the subsequent sequential multiple linear regression analysis was employed to predict WB in postmenopausal women from %BFM, HGS and T-scorert. HGS was the highest predictor of WB(*t* = 2.203; β=0.3046) in postmenopausal women (Table 5).

**Table 5:** The Estimated Regression Coefficients for the two explanatory variables Entered into the Model for postmenopausal women (*N* = 50)

	Zero-Order *r*					
Variables	Tscorert	HGS	WB	β	t	*b*
HGS		1	0.303*	0.3046*	2.203	0.1578
Tscorert	1	0.01	−0.237*	− 0.2386*	−1.69	− 1.3532
Mean	2.19	27.57	10.88			
*SD*	0.29	3.18	5.80	R^2^ = 0.1489		

*p < .05; Tscorert = Standardized bone mineral density score for right big toe; HGS = Handgrip strength; %BFM = Percentage body fat mass; WB = Walking balance; Multiple *R*^*2=*^= 0.1489; Adjusted Multiple R^2^ = 0.1126; std. error of estimate = 5.3492; F (2, 47)= 4.11; *p* = 0.023*; a = 5.4805. In postmenopausal women, the regression Model equation for WB = 5.4805 + 0.1578(HGS) + (− 1.3532)Tscorert

**Table 6:** Three explanatory variables related to walking balance in postmenopausal women (*N* = 50)

	Zero-Order *r*				β		*b*
Variable	%BFM	HGS	Tscorert	WB			
Tscorert				−.24	−.24		−1.3481
HGS			0.01	.30	.30		. 1568
%BF		0.04	−0.02	.06	.04		. .0406
					Intercept =		4.4452
Mean	26.22	27.57	2.19	10.88			
*SD*	6.05	3.18	0.29	5.80	*R*^*2*^ =	. .1506	

Tscorert = Standardized bone mineral density right big toe; HGS = Handgrip strength; %BFM = Percentage body fat mass; WB = Walking balance; Adjusted Multiple **R**^**2**^ = 0. 0953, Std. Error of Multiple Estimate = 5.3436; F(3, 49) = 2.72; p = 0. 0552; Regression model equation for Walking balance = 0.0406 (%BF) + 0. 1568 (HGS) + −1.3481 (Tscorert)

**Table 7:** The Estimated Regression Coefficients for the three explanatory variables Entered into the Model for postmenopausal women (*N* = 50)

Predictor	*r*	β	95% CI ρ
HGS	0.303*	0.345*	0.03, 0.54 .03*
% BFM	0. 061	0.061	− 0.22, .33 .67
Tscorert	−0.237	− 0.256	−0.48, 0.04 .10

*indicates significance at *p* < 0.05; Tscorert = Standardized bone mineral density score for right big toe; HGS = Handgrip strength; %BFM = Percentage body fat mass

Based on our earlier predictions, on the first step, the HGS was entered into the model. It was significantly correlated with WB (R^2^ = 0.09; Adjusted R^2^ = 0.07; Std error of estimate = 5.53; F (1, 48) = 4.86; *p* = 0.03). On the second step (Table 5), T-scorert was entered into the model resulting in significant increase in R^2^ = 0.15; Adjusted R^2^ = 0.11; Std error of estimate = 5.35; F (2, 47) = 4.11; *p* = 0.02). Thereafter, the remaining predictor (i.e. %BFM) was entered into the model (Tables 6 and 7) resulting in no significant increase in *R*^*2*^ = 0.15; Adjusted R^2^ = 0. 10; Std error of estimate = 5.34; F (3, 46) = 2.72; *p* = 0.06. Since the level at which R^2^ reaches a maximum and decreases afterward, would be the regression with the ideal combination of having the best fit and since the full model *R*^*2*^ was not significantly greater than zero when the three predictor variables were combined, the best fit was therefore realized when only two predictor variables - HGS and T-scorert - were combined. The regression model equation for predicting WB in postmenopausal women was therefore given as WB =5.4805 + 0.1578(HGS) + (− 1.3532)T-scorert.

## Discussion

### Relevance of findings to the field

There are differences in body composition between premenopausal and postmenopausal women suggesting a re-compartmentalization of the body. For instance, despite the similarity in the BMI range and category for both groups, the %BFM, FFM, BMI and FM were significantly higher but not the FFM, among postmenopausal women than the premenopausal women. Similar findings have been reported elsewhere by previous authors [2, 3] .Based on the *p*-values, it was projected that postmenopausal changes might have a more profound impact on the %BFM compared to FM. Therefore, fat re-distribution might be more sensitive to postmenopausal changes than the overall FM [42, 43] Invariably, fat adiposity in postmenopausal women might be an important difference that may have greater clinical implications for health. Already, fat adiposity has been strongly associated with C-reactive protein - a major biomarker of systemic inflammation, which lies on the biological pathways to some non-communicable diseases [44, 45] that may affect bone health.

Importantly, a significantly higher %BFM could be an indication of a relatively lower FFM or lean muscle mass if the body mass was constant. This is plausible since BM = %BF + FFM and therefore translates to a proportionate or relative weakening of the skeletal musculature required for ambulation and balance control. Given that postmenopausal changes are age-related [8] it was expected that age might likewise have significant negative effects on HGS and therefore contribute to a weakened musculature in postmenopausal women. On the contrary, ANCOVA analysis revealed that the main predicated effects of age on HGS was not significant likewise its interaction effects with %BFM. However, %BFM significantly affected HGS. Therefore, differences in the body composition, especially %BFM, in postmenopausal women compared to premenopausal women might partly explain why the HGS of the former was significantly weakened than the later. Since the skeletal muscle is part and parcel of the locomotor apparatus [2, 17, 18] ,the significantly weakened muscle strength may also significantly impact WB [2] and may help to explain its variance in postmenopausal women.

Muscle atrophy has been reported in postmenopausal women [46] and may partly explain the significantly weakened HGS observed in this population relative to premenopausal women, in this study. The significantly lower HGS (muscle strength) combined with significantly higher BMI (load) in postmenopausal women compared to premenopausal women, may suggest a relative increase in limb loading. This may lead to a misrelation between the load and available muscle strength, which might create a more difficult operational condition for the locomotor apparatus.

### Implications for care teams and policymakers

Correlation and multiple regression analyses were conducted to examine the relationship between body composition (%BFM, FFM BMI and FM), muscle strength (HGS), BMD (T-scorert) and WB. The HGS + %BFM + T-scorert predictive model, produced R^2^ = 0.0467; F (7, 42)=1.34; *p* = 0.256 *R*^*2*^ = 0.15; Adjusted R^2^ = 0. 10; Std error of estimate = 5.34; F (3, 46) = 2.72; *p* = 0.06. This model could only explain 10% of the variance in WB when adjusted for other confounders. The model with the best fit is HGS + T-scorert model, which produced R^2^ = 0.15; Adjusted R^2^ = 0.11; Std error of estimate = 5.35; F (2, 47) = 4.11; *p* = 0.02 and explained 11% of the variance in WB, when adjusted for the confounding variables. Nevertheless, the proportion of variance is small suggesting that other factors might also explain the variance in WB**.** Overall, the BMD is not a good predictor of WB in postmenopausal women, hence those ratings had little to offer compared to HGS in relation to WB, in the predictive model. Therefore, interventions that increase BMD may predispose to fail without a complementary muscle strengthening program. This view is reasonable and is supported by previous evidence [47, 48]. It was recognized, however, that the BMD assessment may provide the clinicians with valuable information on the potential effects of menopause on bone health which is not considered in the analysis reported here. It must also be considered that the postmenopausal women may gain valuable information about bone health during BMD assessment - an information which may help them better re-evaluate their subsequent lifestyle including physical activity level, medication and nutrition.

The broader implications of these findings could also have translational relevance in clinical practice and management of osteoporosis or fall in postmenopausal women. It will mean that when the BMD of postmenopausal women is improved using medications/nutrition, there must be a complimentary build-up of muscle strength using relevant therapeutic exercises to enhance WB. This is important, because only the HGS (muscle strength) out of the three explanatory variables, is significantly and positively correlated with the WB. It indicates that those with higher mean scores on HGS may tend to have higher or improved WB. Consequently, interventions targeted at improving the muscle strength (HGS) may improve limb loading likewise WB in postmenopausal women.

### Strength and limitations of study

The present study holds strength in the diverse nature of the sample. The effects of muscle strength, body composition and BMD on WB in postmenopausal women, was simultaneously investigated in a very large and well-described hospital community. The hospital-based design allowed simultaneous presentation of findings from health and non-health workers with diverse lifestyles, views, opinions and experiences. Moreover, many studies have been done on menopause in this hospital community (i.e. the UNTH Enugu) [2, 21], and therefore allows for contextual interpretations of the findings relative to previous studies. The application of appropriate statistical control of the effects of covariates, such as age, on muscle strength relative to %BFM, may minimize the threats to the validity of its findings. However, the self-reported period of menopause is certain to include recall error or even bias which may affect the accuracy of the information provided by the participants. In addition, the cross-sectional nature of the study makes it difficult to infer causality between muscle strength, body composition, BMD and WB, respectively. Over time, as a woman transits from premenopausal to postmenopausal status, longitudinal observations on muscle strength, body composition, BMD and WB would be possible, but were not explored in this study. In spite of these limitations, the strengths of the study suggest that it has both scientific and practical implications.

## Conclusion

Muscle strength is the highest predictor of WB and has a significant positive regression weight, indicating that postmenopausal women with greater muscle strength were expected to have better WB. The BMD was not significantly lower in postmenopausal women than premenopausal women, but the variance (R^2^) change was significant from HGS model to HGS + BMD model. However, the proportion of variance in WB explained by this model is small. Invariably other factors, such as psychosocial, physiological and sociological variables, apart from, musculoskeletal health may be necessary to improve WB and mitigate fall risk in postmenopausal women. A wholistic management may, therefore, entail broader clinical considerations for other options such as physical exercises, counseling, and psychotherapy, in addition to medication, nutrition and musculoskeletal health.

## Abbreviations

% BFM: Body fat mass
ANCOVA: Analysis of covariance
BM: Body mass
BMD: Bone mineral density
BMI: Body mass index
DEXA: Dual Energy X-Ray Absorptiometry
FFM: Free mass
FM: Fat mass
Ht: Height
NGS: Handgrip strength
OLS: Ordinary least square
R^2^: variance
T-scorert: T-score of the right big toe
UNTH: University of Nigeria Teaching Hospital
W: Weight
WB: Walking balance
